# 

**Published:** 1915-02

**Authors:** 


					﻿Aim high^tho thou have time* for bgt a single line,
Not failure but low aim is crime?5 .
Vok XXX
FEBRUARY, 1915
No. 8
0 iiIBMilM
■EXf: MEDICAL jpuErgM
PUBLISHED MONTHLY AT AUSTIN, TEXAS, BY
MRS. F. E. DANIEL, - - - Publisher and Managing Editor
PRINTED By the von BOECKMA NN-JONES co.
Subscription, S1.00 a Year, in Advance. -	-	- L W^devonies,w Seats
Entered at the PostofHce at Austin, Texas, as second chips rnuij mattor-.
■
»
>
II
a
&
I
				

